# Foraging across the life span: is there a reduction in exploration with aging?

**DOI:** 10.3389/fnins.2013.00053

**Published:** 2013-04-17

**Authors:** Rui Mata, Andreas Wilke, Uwe Czienskowski

**Affiliations:** ^1^Center for Adaptive Rationality, Max Planck Institute for Human DevelopmentBerlin, Germany; ^2^Evolution and Cognition Lab, Department of Psychology, Clarkson UniversityPotsdam, NY, USA; ^3^Center for Adaptive Behavior and Cognition, Max Planck Institute for Human DevelopmentBerlin, Germany

**Keywords:** aging, cognitive ability, exploitation, exploration, foraging, life span, search

## Abstract

Does foraging change across the life span, and in particular, with aging? We report data from two foraging tasks used to investigate age differences in search in external environments as well as internal search in memory. Overall, the evidence suggests that foraging behavior may undergo significant changes across the life span across internal and external search. In particular, we find evidence of a trend toward reduced exploration with increased age. We discuss these findings in light of theories that postulate a link between aging and reductions in novelty seeking and exploratory behavior.

## Introduction

Any search or foraging act represents a balance between exploration and exploitation: One the one hand, one must search or explore the environment in order to find and learn about desired resources; on the other hand, one must exploit those resources in order to accumulate gains. Consequently, striking a balance between exploration and exploitation is the key to successful foraging. But does aging impact the control of exploration-exploitation trade-offs?

There are two hypotheses that link aging to reductions in exploratory tendencies. First, there is a functional *adaptivity* hypothesis that can be derived from the principle that any agent faced with an exploration-exploitation trade-off may be well advised to initially explore and later exploit its environment. Exploration is an adaptive first step because it allows one to acquire information about the environment that will later lead to successful exploitation (Sutton and Barto, 1998). When such a general principle is translated into an agent's life span, one can predict that reducing exploration with increased age/experience is adaptive (Eliassen et al., 2007). In other words, a life-history trade-off may be expected, involving significant risk taking and exploration early in life and increased exploitation later in life when proximity to death is near and the advantages of exploring the environment for future exploitation are smaller (Carstensen, 2006; Eliassen et al., 2007; Wang et al., 2009). Second, there is a mechanistic *cognitive decline* hypothesis that links aging to changes in exploration-exploitation that does not necessarily imply that such changes are adaptive. Namely, changes in dealing with exploration-exploitation tradeoffs may be brought about by deleterious effects of aging on the mechanisms that control the pursuit of novelty and exploration (Duzel et al., 2010). In sum, both adaptive and mechanistic hypotheses predict that aging should be associated with reductions in exploratory tendencies. But what evidence is there of age differences in novelty seeking and exploratory behavior?

Research on humans suggests that openness and novelty seeking declines over the life span as measured by self-report (Roberts et al., 2006; Lucas and Donnellan, 2011). Also, there is evidence for reduced exploration in the social domain, as indexed by motivation to pursue new social relations (Lang and Carstensen, 2002), and the consumer domain, as indexed by pre-decisional information search (Mata and Nunes, 2010). Risk taking could be considered another component of exploration but the patterns regarding the impact of aging on risk taking are mixed, with evidence from population statistics such as the prevalence of violent crime suggesting reductions in risk taking throughout adulthood but laboratory evidence showing inconsistent effects (Mata et al., 2011). Research on animal models supports the idea that aging is associated with changes in novelty seeking and exploratory behavior in some species. Regarding non-human primates, there is evidence for reductions in attentiveness to a novel task (Kendal et al., 2005) and ratings of extraversion and openness with increased age (Weiss et al., 2007; King et al., 2008). Also, there is evidence for reduced exploration with increased age in some types of wasps (Thiel et al., 2005), fish (Yu et al., 2006), and rats or mice (Lalonde, 2002). All in all, the evidence listed above suggests that aging may be associated with changes in novelty seeking and exploratory behavior but evidence is still lacking regarding possible underlying mechanisms.

We suggest that developmental research may profit from investigating foraging behavior to understand the link between aging and exploratory tendencies. Foraging is a crucial adaptive problem that presents a clear trade-off between exploration and exploitation (Stephens, 2008) and one that spans many domains, including the search for tangible resources such as food (Gurven et al., 2006), or, alternatively, abstract ones such as information in the external world (Pirolli and Card, 1999), or memory (Hills et al., 2012).

Crucially, there has been considerable interest and progress of late in understanding the cognitive and neural basis of foraging decisions (Pirolli and Card, 1999; Cohen et al., 2007; Payne et al., 2007; Hills et al., 2010, 2012; Hayden et al., 2011; Kolling et al., 2012). One important realization from this line of work is that the mechanisms involved in foraging decisions may be domain-general and thus apply to both internal and external search. First, there are strong similarities in the search mechanisms used across tasks, for example between external search (Hutchinson et al., 2008) and search from memory (Wilke et al., 2009). Second, there is evidence for cross-domain priming; exploration in a visual-spatial search task primes exploration in a lexical task (Hills et al., 2010). Third, there is evidence for domain-general neural mechanisms underlying search processes that are likely shared by different species (Daw et al., 2006; Hills, 2006; Cohen et al., 2007; Hayden et al., 2011). For example, Cohen et al. have posited an important role for catecholamines, such as norepinephrine and dopamine in balancing the choice between choosing (exploiting) old rewards and switching to (exploring) new ones. But how do such systems that likely underlie foraging processes change as a function of aging?

There is evidence for considerable age-related cognitive decline in primates due to structural and functional brain changes (Arnsten and Goldman Rakic, 1985; Hof and Morrison, 2004). For example, prefrontal brain areas underlying exploration-exploitation decisions during foraging (Daw et al., 2006; Hayden et al., 2011; Kolling et al., 2012) are particularly affected by aging (West, 1996). Age-related structural deterioration of the substantia nigra and ventral tegmental area seem to have implications for overall catecholaminergic neuromodulation (Arnsten, 1998; Li et al., 2001). Given the role of catecholamines in modulating learning, novelty seeking, and explorative behavior (Hills, 2006; Cohen et al., 2007; Doya, 2008; Eppinger et al., 2011), age-related deficits in catecholaminergic modulation could be expected to lead to age differences in foraging. In sum, neural and cognitive theories suggest that the control of exploration-exploitation trade-offs is domain-general and, consequently, age-related change should affect foraging spanning external and internal representations.

Results from two studies are compatible with the idea that aging is associated with reduced exploratory behavior in external foraging (Mata et al., 2009; Louâpre et al., 2010). Louâpre et al. asked an age-heterogeneous sample (18–57) to forage for treasure chests in various domes scattered in a virtual meadow and found that older participants tended to stay longer at the current resource patch (i.e., dome) relative to younger ones. Similarly, Mata et al. asked younger and older adults to forage for fish in a sequence of virtual ponds and found that older adults tended to search longer in a given pond compared to younger adults, suggesting that older adults may be less willing to explore new resource patches (Mata et al., 2009). One study investigated age differences in information foraging by asking younger and older adults to find words from word puzzles with the goal of maximizing the total number of words found within a limited time period (Chin et al., 2012). The results suggest that older adults were more likely to stay with a particular puzzle relative to younger adults and that the frequency of switching between puzzles—one possible index of exploration—was negatively related to higher fluid abilities.

**Figure 1:**
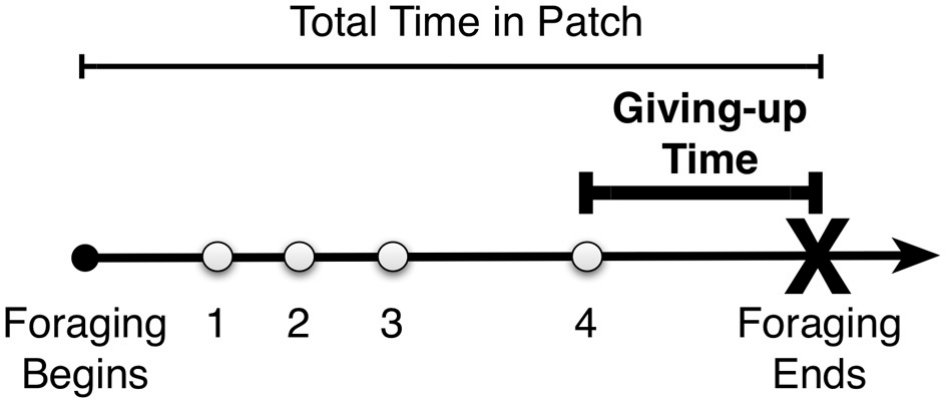
**Example time line of foraging in a patch.** The participant decides to leave the patch some time after finding the last of four items—the giving-up time.

In the following, we aim to contribute to further documenting the scope of age differences in foraging. We hypothesize that to the extent that aging leads to changes in domain-general neural and cognitive mechanisms responsible for foraging, we should find similar patterns of age differences across tasks. We test this general prediction by presenting data from two tasks used previously to investigate foraging in external and internal representations (see Table 1 for a description of the two tasks and associated references). The first set of data stems from the Mata et al. (2009) study described above that asked younger and older adults to forage for fish in virtual ponds and assessed their foraging policies as a function of the time delay between ponds (patches). The results suggest that younger and older adults are similarly sensitive to time delays and are thus adaptive foragers in what concerns the travel costs between resource patches. In the results below, we go beyond the original analyses by Mata et al. (2009) by analyzing individuals' giving-up times, that is, the time between the last resource found in a patch and the decision to leave the patch and explore a new one (see Figure 1). Giving-up times have been suggested to be reliable measures of exploratory tendencies (Dougherty and Harbison, 2007; Harbison et al., 2009) but to our knowledge there have been no investigations of age differences in giving-up times. Furthermore, we present data from two unpublished experiments on search in memory, which used an analogous design to the one in Mata et al. (2009) but asked younger and older adults to search for word solutions in memory.

**Table 1:** **Foraging tasks**.

**Task**	**Task description**	**Reference(s)**
Fishing task	Participants are presented sequentially with ponds (i.e., patches) in which they forage for fish, and can decide on how long to stay at each pond. All ponds appear equal, but the number of fish in each varies according to the underlying resource distributions (e.g., negative binomial). Once participants decide to switch between ponds they incur a time delay (i.e., travel time) in which they experience a bouncing ball.	Hutchinson et al., 2008; Mata et al., 2009
Word Puzzle task	Participants are presented sequentially with meaningless sequences of letters, from which they can generate meaningful words from their mental lexicon, and can decide on how long to work on a given sequence. Analogously to the Fishing task, participants experience different types of patch quality distributions and experience time delays between letter sequences in which they observe a bouncing ball.	Wilke et al., 2009

In addition, we link giving-up times in the different experiments to a measure of fluid ability, and for a subset of our data, other covariates, to test hypotheses concerning the underlying causes of age differences in exploratory tendencies. The adaptive hypothesis of age-related reductions in novelty seeking and exploration emphasizes the role of opportunity costs. Consequently, motivational variables such as future time perspective (Lang and Carstensen, 2002; Carstensen, 2006) and maximizing (Dougherty and Harbison, 2007) may index the subjective value of exploration and thus be linked to giving-up times. In contrast, the cognitive decline hypothesis of age-related reductions in novelty seeking and exploration suggests that age-related cognitive decline is the main factor underlying reductions in exploratory behavior, and thus could be related to measures of fluid cognitive ability (Duzel et al., 2010). In sum, investigating the correlation between exploratory tendencies and individual difference measures could be informative regarding the factors responsible for age differences in exploration-exploitation.

## Materials and methods

### Participants

**Figure 2:**
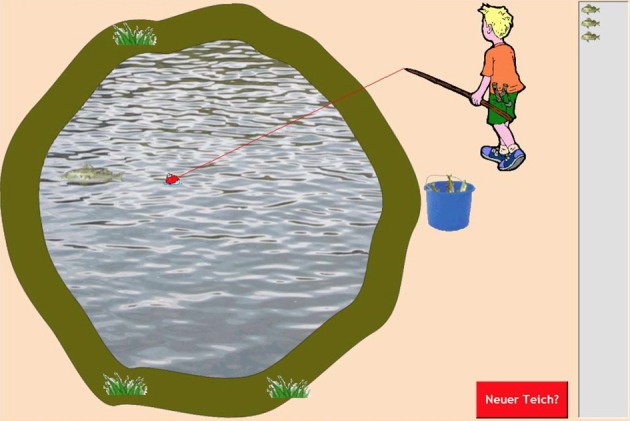
**Screenshot of the Fishing task (Czienskowski, 2005a).** The fisherman is moving the float at the end of the fishing line toward a fish that appeared on the left-hand side of the pond. Three fish have already been caught at the current pond (see resource stack on the right side). Subjects can choose to move to the next pond at any time by hitting the red switch patch button (lower right). Hitting the switching button will let the fisherman walk off the screen, initiate a waiting period in which a bouncing ball animation is shown (i.e., the travel time between subsequent ponds), and ends with the fisherman walking back onto the screen to a new pond (with a centered float, no fish showing in the bucket, an emptied resource stack, and the pond redrawn with different pond margins and vegetation around it).

**Figure 3:**
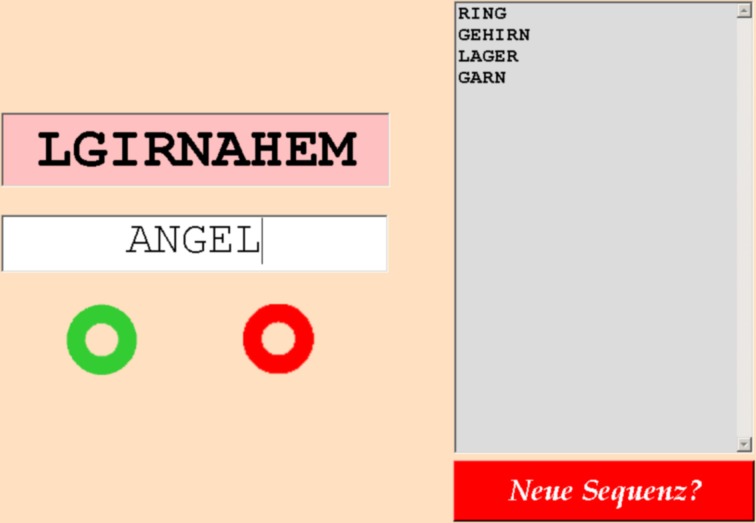
**Screenshot of the Word Puzzle task (Czienskowski, 2005b).** German singular noun word solutions to a letter sequence (here, LGIRNAHEM) are typed into the entry field (here, ANGEL). Subjects receive feedback on if their solution is correct (green circle lights up) or incorrect (red circle lights up). All valid solutions that have been generated so far appear on the word stack (right side). Subjects can choose to move to the next letter sequence at any time by hitting the red switch sequence button (lower right). Hitting the switching button will initiate a waiting period in which a bouncing ball animation is shown (i.e., the travel time between subsequent letter sequences) followed by the appearance of a fresh letter sequence (with the entry field cleared and the word stack emptied).

All participants gave written informed consent before participating in the studies reported below. All experiments were conducted at the Max Planck Institute for Human Development, Berlin, Germany, and approved by the Ethics Board of that institution. We report data from four experiments, two involving the Fishing task and two the Word Puzzle task (see Figures 2, 3). The data from the Fishing task stems from two separate experiments originally reported in Mata et al. (2009). The two experiments differed in the initial instructions given to participants: The first experiment provided no explicit strategy instruction, while the second experiment instructed participants to use an incremental foraging strategy (cf. Mata et al., 2009). Below, we aggregate the samples from the two experiments because participants' foraging behavior was very similar across experiments. Consequently, the Fishing task data set consists of 150 participants (75 younger and 75 older adults). Participants in the Fishing Task were paid a fee for their participation (€10 per h), plus a bonus relative to how many fish they caught (€0.10 per fish).

Concerning the Word Puzzle task, the data stem from two previously unpublished experiments that differed in the payment scheme. In the first study, 60 participants (30 younger adults and 30 older adults) were paid a fee for their participation (€10 per h), plus a bonus relative to how many words they produced (€0.10 per word). In the second study, 99 participants (49 younger and 50 older adults) were paid a fee for their participation (€10 per h), plus a bonus relative to how many words they produced (€0.10 per word) but also were penalized for incorrect submissions (€0.10 per incorrect submission). As expected, the participants in the two studies differ in their error rates, with participants making fewer incorrect submissions in the experiment that penalized errors, but otherwise the pattern of results, in particular age differences in giving-up times, was similar across experiments and we combine the two samples for the analyses below.

Table 2 presents relevant participant characteristics. All participants completed a crystallized intelligence test (Lehrl, 1999), and a fluid ability test (Wechsler, 1981). In addition, a subset of participants that participated in the second experiment involving the Word Puzzle task (*N* = 99) completed a number of questionnaire measures that we reasoned could be related to exploratory behavior, including risk-taking in the investment and gambling domain (Weber et al., 2002), maximization tendencies (Schwartz et al., 2002), and future time perspective (Lang and Carstensen, 2002).

**Table 2:** **Participant characteristics**.

**Characteristic**	**Fishing task**		**Statistic**		**Word Puzzle task**		**Statistic**	
	**Younger**	**Older**	***t*_(148)_**	***p***	**Younger**	**Older**	***t*_(157)_**	***p***
*N*	75	75	–	–	79	80	–	–
Sex (Male)	30 (40%)	33 (45%)	–	–	32 (41%)	39 (49%)	–	–
Age	24.1 (3.2)	70.6 (4.0)	–	–	24.6 (3.4)	69.6 (4.0)	–	–
Vocabulary	30.4 (2.4)	33.0 (2.6)	6.27	<0.001	31.1 (2.6)	32.5 (2.3)	3.62	<0.001
Processing speed	59.8 (8.1)	42.1 (9.1)	12.07	<0.001	65.1 (11.3)	44.4 (8.7)	12.99	<0.001

### Procedure

Before starting any of the experiments, participants were asked to put aside any devices (e.g., watches, cell phones) that could be used as external timekeepers. For all experiments, participants received instructions on the computerized display and experienced a training phase identical to the main experiment that was used to help participants familiarize themselves with the apparatus and task. In the Fishing task, participants received instructions on how to use a touch screen to catch fish and leave patches (Hutchinson et al., 2008; Mata et al., 2009), and in one experiment instructed to use an incremental foraging strategy (cf. Mata et al., 2009). In the Word Puzzle task, participants were instructed on how to use the mouse and keyboard to type solutions and leave patches (cf. Wilke et al., 2009). In addition, participants were informed about the restraints in the type of words that could be submitted and answered a 25-item multiple-choice quiz on submission rules as a comprehension check (Wilke et al., 2009). The main goal of all experiments was to test for sensitivity to time-delay between patches, consequently, each participant completed two versions of the Fishing or Word Puzzle tasks in which the travel time between resource patches (i.e., ponds, word puzzles) were either short (15 s) or long (35 s). Participants then foraged in each version of the task for a limited time (40 min) and took a short break between the two versions. After the foraging experiments, participants answered some questions about the task, and completed the additional individual differences measures.

## Results

Our analyses had two goals. First, we were interested in determining whether we would find systematic age differences in giving-up times in both external and internal foraging. Second, we aimed to link giving-up times to a number of individual differences measures to test for potential links between reductions in exploratory tendencies and individual differences in cognitive ability, risk taking, maximization tendencies, and future time perspective.

### Giving-up times

**Figure 4:**
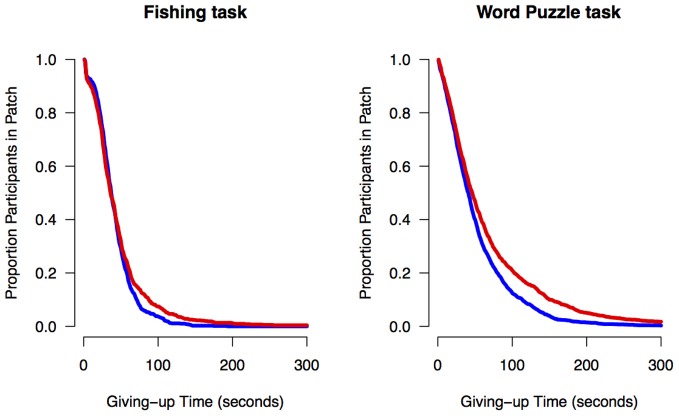
**Proportion of younger (blue line) and older adults (red line) in a patch as a function of time in the Fishing and Word Puzzle tasks**.

We used the Cox proportional hazard regression model to quantify age effects on giving-up times, that is, the time between the last capture in each patch and the decision to leave the patch. Cox regression is a method designed to analyze survival data for which the outcome variable is the timing of an event (Cox, 1972). The Cox regression analysis consisted of regressing dummy coded variables for age group (younger adults = 0, older adults = 1) on individuals' giving-up times. The regressions showed an effect of age group for both the Fishing task, exp(B) = 0.91, *z* = 2.04, *p* = 0.04, and Word Puzzle task, exp(B) = 0.87, *z* = 3.71, *p* < 0.001, suggesting that older adults tended to stay longer in a patch relative to younger adults. These results remained significant when controlling for the number of captures per patch, suggesting that age-related differences in giving-up times are not a function of overall foraging performance. For visualization purposes, we plotted the percentage of participants that remained in a patch as a function of time since the last capture in a patch. As can be seen in Figure 4, a higher proportion of older adults tended to stay in the patch relative to younger adults since the last capture, which could be interpreted as an age-related decrease in exploration.

### Giving-up times and individual difference measures

We assessed the link between individual differences in exploratory tendencies and fluid abilities by correlating individuals' mean giving-up time scores on individual difference measures. We computed the correlations separately for the two age groups to avoid biasing our results by mean age group differences in the individual difference measures (Hofer and Sliwinski, 2001). Table 3 presents all computed correlations coefficients. We found a significant negative correlation between fluid abilities and giving up times for the older sample that completed the Word Puzzle task but this was the only significant effect that emerged from these analyses.

**Table 3:** **Correlations between giving-up times and individual difference measures**.

**Characteristic**	**Fishing task**		**Word Puzzle task**	
	**Younger**	**Older**	**Younger**	**Older**
Processing speed	0.13	0.02	−0.17	−**0.28**
Vocabulary	0.08	−0.12	−0.06	−0.09
Maximizing	–	–	−0.15	0.04
Decisiveness	–	–	0.01	0.05
Investment	–	–	0.08	0.08
Gambling	–	–	−0.17	0.16
Future time perspective	–	–	0.25	−0.04

*Note: Significant correlation with p < 0.05 in bold*.

## Discussion

Functional and mechanistic accounts of aging suggest that there may be a reduction in exploratory tendencies with advanced age. We presented results from two foraging tasks, the Fishing and Word Puzzle tasks, suggesting that older adults show longer giving-up times relative to younger adults, a measure that has been suggested to represent reliable individual differences in exploratory tendencies (Harbison et al., 2009). Our results are in thus line with views suggesting that aging is associated with a reduction in exploratory tendencies in both external and internal search.

Past work on the life span development of human foraging has focused on the interplay of physical prowess and experience in determining foraging, in particular, hunting success (Walker et al., 2002; Gurven et al., 2006). An important conclusion from such work is that physical decline can account for a large portion of age-related decline in performance, a general result that matches similar findings from the non-human literature (MacNulty et al., 2009; Zimmer et al., 2011). Our work emphasizes that exploratory tendencies may also change systematically across organisms' life spans and thus raise the interesting question of whether reductions in exploratory tendencies can be also partly responsible for age-related declines in foraging performance. Naturally, changes in exploratory tendencies will have different impact depending on the structure of the task (Mata et al., 2012). Consequently, it will be important to consider task characteristics to understand whether reduced exploration can lead to changes in foraging performance.

Our results are not conclusive regarding the mechanisms underlying age differences in giving-up times. We conducted exploratory analyses of the link between giving-up times and individual differences measures, including measures of fluid abilities, future time perspective, maximizing, and risk taking. The rationale for using these measures was that different explanations of reductions in exploratory tendencies with aging suggest different underlying mechanisms and, hence, covariates. Adaptive explanations of the link between aging and exploration suggest that reductions in exploratory tendencies may be accompanied by decreased subjective value given to exploration, which may be indexed by measures such as future time perspective (Lang and Carstensen, 2002) or maximizing (Harbison et al., 2009). We did not find a link between motivational variables and exploratory tendencies, and therefore our results do not favor an adaptive explanation linking reductions in exploration to age differences in motivation. Alternatively, a mechanistic hypothesis linking aging and exploration is that the deleterious effects of aging in cognitive ability are accompanied by age-related reductions in novelty and exploration because they rely on similar neural substrates (Duzel et al., 2010). We found a significant correlation between exploration and cognitive ability for only one of our samples of participants, which provides some, albeit admittedly weak evidence for the latter hypothesis. In sum, these results suggest that more work has to be conducted to identify links between foraging and individual differences in cognitive and personality characteristics.

Our studies have a number of limitations. First, we did not consider a number of other potentially relevant variables that may underlie age differences in exploratory tendencies. For example, human and animal research suggests that aging may be related to deficits in time estimation (McCormack et al., 2002) and that time estimation is a good indicator of fronto-striatal integrity (Wild-Wall et al., 2008). Similarly, there are known age differences in time valuation (Samanez-Larkin et al., 2011). Some have suggested that tonic dopamine levels in striatum encode the subjective value of time (Niv et al., 2006). According to this hypothesis, age-related decreases in tonic dopamine levels would result in time being valued less, leading to a potential reduction in the subjective costs of a delayed reward. In support of this idea, reductions in dopaminergic markers have been reported in aged rats, which show less time discounting (Simon et al., 2010). In sum, age-related cognitive decline may be linked to deficits in time estimation or reduced time valuation, which in our task could have led to longer giving-up times. Future studies that measure both individuals' time estimation and temporal discount rates would permit shedding light on the link between such variables and giving-up times.

Another limitation of our work is that we cannot exclude that the observed age differences in giving-up times results from age differences in learning abilities. Given the well-documented age-related deficits in learning it is possible that older adults would simply need more time to improve their performance (Eppinger et al., 2011). Future work that provides additional learning opportunities to older participants could thus be important to evaluate the role of learning in age-differences in exploration-exploitation. More generally, future work may be more successful in targeting the causes underlying age differences in foraging by making use of direct manipulations or longitudinal designs. For example, taxing cognitive resources with a secondary cognitive task could test the idea that fluid cognitive ability differences are crucial to adjusting giving-up times. In turn, longitudinal designs would allow assessing whether the development of age-related cognitive decline tracks that of exploratory tendencies.

Finally, one limitation of our work was the reliance on a reaction-time measure-giving-up times-to describe age differences in exploratory behavior. Although giving-up times have been suggested to capture reliable individual differences in search (Dougherty and Harbison, 2007), any reaction-time based measure poses interpretational problems regarding exploratory tendencies with aging due to overall age differences in motor and cognitive speed (Salthouse, 1996). One avenue for future work would be to use other tasks that allow the use of alternative dependent measures that are not based on reaction-times to capture exploratory tendencies. Suitable tasks may include information search tasks that use switching between options (Daw et al., 2006), cues (Hills et al., 2013), or problems (Chin et al., 2012) as indicators of exploration.

We have suggested that developmental research may profit from considering age differences in foraging behavior to understand the impact of aging on exploratory tendencies. However, research on the cognitive mechanisms underlying foraging may in turn profit from a developmental perspective. In particular, there is tension between attempts to explain perceptual search in light of optimal foraging theories (Cain et al., 2012) and results suggesting that humans are not optimal foragers in more complex tasks (Hutchinson et al., 2008). One interesting avenue for future work that could further elucidate the generality of foraging mechanisms would be to test for differential aging effects on exploration in different domains, such as visual search (Cain et al., 2012), search in space (Hills et al., 2010), memory (Hills et al., 2012), or information (Pirolli and Card, 1999). Understanding foraging processes across domains and populations surely needs and deserves additional exploration.

### Conflict of interest statement

The authors declare that the research was conducted in the absence of any commercial or financial relationships that could be construed as a potential conflict of interest.
